# Supplementary material for: MetaRibo-Seq measures translation in microbiomes
Source: Nat Commun. 2020 Jun 29;11:3268. doi: 10.1038/s41467-020-17081-z (PMC7324362; doi:10.1038/s41467-020-17081-z)
Supplement: Supplementary file 10 — Supplementary Data 7 [file 41467_2020_17081_MOESM10_ESM.zip › File2/Confidence_VeryHigh_Taxonomy/188100_out.krona.html]

Javascript must be enabled to view this page.

members
magnitude
magnitudeUnassigned
count
unassigned
taxon
rank

188100\_out

5

2
5
superkingdom

1224
phylum
5

1236
class
5

order
5
91347

543
family
1
5

SRS140492\_contig\_number\_contig-100\_6373.164786

561
4
genus

species
4

SRS049402\_contig\_number\_12875SRS078176\_contig\_number\_4552SRS146888\_contig\_number\_11310SRS147022\_contig\_number\_7462
562
